# Analytical First Derivatives of the SCF Energy for the Conductor‐Like Polarizable Continuum Model With Non‐Static Radii

**DOI:** 10.1002/jcc.70099

**Published:** 2025-04-24

**Authors:** Lukas Wittmann, Miquel Garcia‐Ratés, Christoph Riplinger

**Affiliations:** ^1^ Mulliken Center for Theoretical Chemistry University of Bonn Bonn Germany; ^2^ FAccTs GmbH Cologne Germany

**Keywords:** cpcm, density functional theory, draco, gradient, implicit solvation, solvation

## Abstract

Within this work, we present the derivation and implementation of analytical gradients for the Gaussian‐switching (SwiG) Conductor‐like Polarizable Continuum Model (CPCM) with general nuclear coordinate‐dependent non‐static radii used for the creation of van der Waals‐type cavities. This is done using the recently presented dynamic radii adjustment for continuum solvation (draco) scheme. This allows for efficient geometry optimization and reasonable numerical Hessian calculations. The derived gradient is implemented in ORCA, and therefore is easily applicable. The derivation and implementation is validated by comparing analytical and numerical gradients and testing geometry optimizations on a diverse test set, including small organic compounds, metal‐organic complexes, and highly charged species. We additionally test the continuity of the potential energy surface using an example where very strong changes in the radii occur. The computational efficiency of the derived gradient is investigated.

## Graphical abstract

Derivation and implementation of the analytical gradients for the iSwiG CPCM model with general coordinate‐dependent radii. This is done using van der Waals cavities in combination with the DRACO scheme.

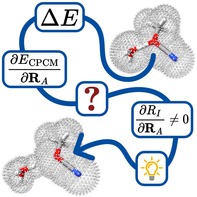

## 1 Introduction

Understanding molecular behavior in solution is crucial for many fields, from chemical synthesis to material science to biological and pharmaceutical research. However, the field of solvation models remains inadequately resolved, with no universally applicable high‐level method currently available [1, 2, 3]. All well‐performing quantum mechanical (QM) models rely heavily on empirical parameters, which limits their general applicability and performance for applications and systems away from the training data [4, 5, 6, 7, 8]. In computational chemistry, there are two main approaches to account for solvent effects. The first of them, involves implicit solvation models, which try to capture the impact of a solvent on a solute without explicitly representing the individual solvent molecules [9]. These models often approximate the interaction with the solvent as an interaction with a (polarizable) dielectric continuum [10, 11, 12]. Popular models are the conductor‐like screening model (COSMO) and the conductor‐like polarizable continuum model (CPCM) [13], or the domain‐decomposition variants ddCOSMO [14, 15, 16, 17, 18] and ddPCM [19, 20, 21]. These approaches, however, only account for electrostatic interactions. To improve the results, some more sophisticated models are based on the aforementioned electrostatic treatment, but additionally include terms for non‐electrostatic interactions. Popular choices include the conductor‐like screening model for realistic solvents COSMO‐RS [22, 23, 24] its open‐source implementation openCOSMO‐RS [25], and the SMD solvation model [26, 27]. These more sophisticated methods, like SMD and COSMO‐RS, already show good performance; however, they can still struggle with very polar (anisotropic) solvents and can yield bad performance for strong polar, ionic, or electronically challenging solutes [28, 29]. Also, due to the empiric nature of these models, performance on systems that differ significantly from the training set may not yield good results and can exhibit more pronounced errors [30, 31, 32]. For fast semi‐empirical electronic structure methods, there are also very efficient methods such as the ALPB, GBSA, or CPCM‐X models, that also include non‐electrostatic contributions [33, 34]. The second strategy to model the solvent in QM calculations involves explicit solvation methods, where one includes the solvent molecules directly. This approach requires a comprehensive sampling of the conformational space, which can be achieved using molecular dynamics techniques such as metadynamics [35]. While thorough sampling is always essential, the computational cost increases significantly with the addition of more solvent molecules. Consequently, while classical or semi‐empirical methods can manage the sampling of the potential energy surface (PES), this is computationally too expensive, with high accuracy, for QM calculations [36, 37, 38, 39, 40]. An alternative to explicit and implicit solvation methods are the so‐called hybrid approaches, such as microsolvation, where the system is solvated by a limited number of explicit solvent molecules, while the bulk of the solvent is described via implicit solvation [41]. This can balance accuracy and computational cost. There are various different approaches like the weighted‐random minimum structure approach [42] combined with subsystem density functional theory [43] or the Quantum Cluster Growth (QCG) workflow [44], combined with the conformer‐rotamer ensemble sampling tool, CREST [45, 46, 47]. Together, these workflows generate a comprehensive ensemble of the solvated molecule, exploiting the benefits of both explicit and implicit solvation methods. With such workflows, it is possible to compute, for example, spectroscopic properties, such as IR or RAMAN spectra, including explicit solvent molecules and thus explicit solvent‐solute interactions, at a feasible computational cost [44]. Yet, this approach still provides poor $\Delta G_{\text{solv}}$ values, additionally requiring expert knowledge and attention to detail [48, 49]. Moreover, these approaches are far from being black‐box solutions. Thus, the topic of solvation still represents a central, only partially solved problem in theoretical chemistry [9, 10, 50].

One recent advancement in the field of solvation is the dynamic radii adjustment for continuum solvation (draco) approach [51]. This method aims at providing system‐specific radii that are used for the construction of the cavity that is needed for the electrostatic part of the solvation effects. The radii of the atoms that form the cavity around the solute in an implicit solvation model are very important and heavily determine its performance. This can be seen with the SMD and SMD‐B solvation models [27]. However, the SMD‐B model uses Bondi radii, which yield better performance for ions and $pK_{a}$ values—at the cost of worse performance for neutral species. It is generally very difficult to obtain the optimal effective radii of solutes in solution, as they depend not only on the solute itself but also heavily on the interaction with the solvent [52, 53]. The draco model manages to provide system‐ and atom‐specific radii for many popular implicit solvation models like CPCM and SMD. The use of the draco scheme in combination with CPCM (CPCM+draco) or SMD (SMD+draco) substantially improves the calculated free energies of solvation for both neutral and ionic systems. For instance, for CPCM+draco, the mean absolute error (MAE) of the solvation free energy decreases by 67% ($4.5\;\text{kcal}\;{\mathrm{mol}}^{-1}$) for a large set of neutrals and ionic systems, while for SMD+draco the decrease is of 39% ($1.5\;\text{kcal}\;{\mathrm{mol}}^{-1}$) for ions, and of 16% ($0.2\;\text{kcal}\;{\mathrm{mol}}^{-1}$) for neutrals [51].

For every implicit solvation model, it is crucial to have access to the analytical gradient of the energy w.r.t. nuclear coordinates. In this way, one can optimize the geometry of the solute in the presence of a solvent, which can be very different to its counterpart in the gas‐phase. For instance, amino acids are neutral in the gas‐phase, but can be present as zwitterions in an aqueous solution. For most implicit solvation models, like COSMO, CPCM or SMD, the analytical gradient is available, and implemented in most QM packages. Nevertheless, these implementations usually involve fixed radii for the atoms in the cavity of the solute.

In this work, we derive the gradient of the Self‐Consistent Field (SCF) energy w.r.t. nuclear displacements in the presence of an implicit solvent described by the CPCM model with atomic position‐dependent radii by using the novel draco approach. The gradient is derived in a general manner and can thus be applied to any radii scaling model with only minor adjustments. The final gradient is implemented in combination with the draco model into ORCA [54], tested against the numerical gradient, and demonstrated for some showcases. This will allow the usage of the much improved CPCM+draco model for geometry optimizations and possibly open the way for more dedicated models that could potentially be based on the idea of CPCM+draco.

## 2 Theory

In the following subsections, we will introduce the theory behind draco and CPCM, followed by the derivation of the gradient. At first, the draco scaling scheme will be introduced, followed by the CPCM model and the coupled CPCM‐SCF procedure. For the CPCM model, the Gaussian charge scheme will be used and introduced thereafter.

We will use capital letters I to denote an atom or atom‐centered sphere, and non‐capital letters i to denote points on the cavity Γ with i∈Γ. Vectors will be denoted as bolt non‐capital letters and matrices as bolt capital letters, except for the position of the nuclei (and spheres) where we will use a bolt capital R for better readability. Thus, $R_{I}$ denotes the position of the sphere or atom I, $r_{i}$ the position of the point charge $q_{i}$, and $R_{I}$ the radius of sphere I centered at $R_{I}$.

### 2.1 Draco

**FIGURE 1:**
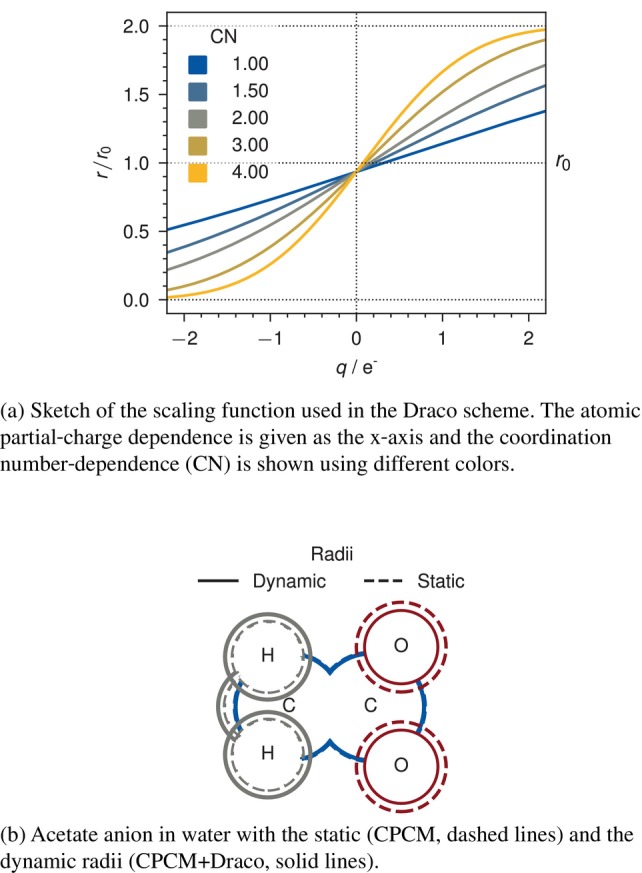
Sketches of the scaling functions (a) and resulting radii for the acetic acid anion in water (b). A phenomenological interpretation can be found in Reference [51].

Within this draco subsection only, the index I is atom specific; $q_{I}$ indicates the atomic partial charge of atom I, and ${\mathrm{CN}}_{I}$ indicates the coordination number of atom I. A sketch of the resulting radii of the draco scheme and an example is given in Figure 1.

Within the draco scheme, the scaled, atom‐specific radii are obtained via

(1) $$R_{I}\equiv R_{I,\text{scal}}=f_{I}R_{I,0}$$

by using a scaling function $f_{I}$ that acts on the static radii $R_{I,0}$. This scaling function is given by

(2) $$f_{I}=\mathrm{erf}\left[a_{Z_{I}}\left(q_{I,\mathrm{eff}}-b_{Z_{I}}\right)\right]+1$$

where, $a_{Z_{I}}$ and $b_{Z_{I}}$ are element‐specific, empirical parameters and $q_{I,\mathrm{eff}}$ is an effective charge. Within draco, $a_{Z_{I}}$ controls the steepness of the scaling and $b_{Z_{I}}$ controls the offset w.r.t. the effective charge. This effective charge is calculated via

(3) $$q_{I,\mathrm{eff}}=q_{I}\left(1+k_{Z_{I}}{\mathrm{CN}}_{I}\right)$$

by using an atomic partial charge $q_{I}$, a coordination number ${\mathrm{CN}}_{I}$, and the empirical parameter $k_{Z_{I}}$, which controls the coordination number dependence of the effective charge. The parameters $a_{Z_{I}}$, $b_{Z_{I}}$ and $k_{Z_{I}}$ can be obtained, for example, like in Reference [51], by parametrization on experimental solvation free energies. The coordination number used is taken from Reference [55] and defined as

(4) $${\mathrm{CN}}_{I}=\sum_{I\neq J}^{N}\frac{1}{f_{1}}\frac{1}{f_{2}}$$

with

(5) $$f_{1}=1+\exp\left[-10\left(\frac{4}{3}\frac{R_{I,\mathrm{cov}}+R_{J,\mathrm{cov}}}{r_{\mathrm{IJ}}}-1\right)\right]\;\text{and}$$

(6) $$f_{2}=1+\exp\left\{-20\left[\frac{\frac{4}{3}\left(R_{I,\mathrm{cov}}+R_{J,\mathrm{cov}}\right)+2}{r_{\mathrm{IJ}}}-1\right]\right\}$$

Atomic partial charges can be obtained via, for example, the Hirshfeld population [56], the electrostatic equilibration charge model (EEQ) [57], or the Charge Extended Hückel (CEH) model [58, 59]. In the original works, Reference [51], it was found that the specific choice among these charge models had only a minor impact on the final results, because any differences were largely absorbed into the respective parameterization. Of these approaches, the EEQ model, being a purely classic model, is the cheapest to evaluate and therefore offers an advantage.

### 2.2 CPCM Equations

Within the polarizable continuum model, the vacuum Hamiltonian $H_{0}$ of the solute is perturbed by an additional operator $V_{s}$, which accounts for the solute's interaction with the surrounding solvent.

(7) $$H=H_{0}+V_{s}$$

This solvation term can be expressed using $N_{q}$ charges, q, which are spread over the solute cavity Γ with their respective positions $r_{i}\in \Gamma$.

(8) $$H=H_{0}+\sum_{i}^{N_{q}}\frac{q_{i}}{∥r_{i}-r∥}$$

Here, $∥r_{i}-r∥$ denotes the Euclidean norm, that is, the distance between points $r_{i}$ and r. The discretization of the cavity surface results in a set of linear equations [52]

(9) Kq=Rv⇔q=Qvwith

(10) $$Q=K^{-1}R$$

where, q are the aforementioned charges, v is the electrostatic potential at the position of the charges, Q is the so‐called *response matrix*. The electrostatic potential is given by

(11) $$v_{i}=\sum_{K=1}^{N_{\text{atoms}}}\frac{Z_{K}}{\left|r_{i}-R_{K}\right|}-\sum_{\mu \nu }P_{\mu \nu }\left\langle \chi_{\mu }\left|\frac{1}{r_{i}-r}\right|\chi_{\nu }\right\rangle$$

which can be separated into nuclear $v_{\mathrm{nuc}}$ and electronic $v_{\mathrm{el}}$ contributions. The form of the matrices K and R, that form the response‐matrix, depend on the choice of the PCM and are described in more detail in, for example, References [11, 60]. Within the conductor‐like PCM, one uses

(12) K=Aand

(13) $$R=-\left(\frac{\varepsilon -1}{\varepsilon +x}\right)I\equiv -f\left(\varepsilon \right)$$

with ε being the dielectric permittivity of the solvent and x=0 for the conductor‐like PCM [39]. The form of the matrix A depends on the discretization scheme used and will be discussed in Section 2.3. Within the conductor‐like PCM, the electrostatic potential due to the point charges cancels the electrostatic potential due to the solute at the cavity surface and allows the charge vector q to be obtained by solving

(14) $$\mathrm{Aq}=-f\left(\varepsilon \right)v$$

The result can then be used to calculate the electrostatic solvation energy of the conductor‐like PCM via one of the equivalent forms of

(15) $$E_{\mathrm{pol}}=\frac{1}{2}q^{⊤}v=-\frac{1}{2}q^{⊤}\mathrm{Aq}=\frac{1}{2}v^{⊤}\mathrm{Qv}$$

### 2.3 Gaussian Charge Scheme

The choice of the discretization scheme defines how the surface charge density is represented and thus defines the form of matrix A. There are many choices; however, the Gaussian Charge Scheme has established itself as it offers greater robustness, for example, it does not suffer from discontinuities in the potential energy surface (PES) compared to regular point charge schemes. This issue is discussed in more detail in, for example, References [11, 60, 61, 62]. The Gaussian charge scheme replaces the point charges typically used with spherical Gaussian functions centered at the positions $r_{i}$ on the cavity (16).

(16) $$g_{i}\left(r\right)=q_{i}{\left(\frac{\xi_{i}^{2}}{\pi }\right)}^{\frac{3}{2}}\exp\left(-\xi_{i}^{2}∥r-r_{i}{∥}^{2}\right)$$

Here, $q_{i}$ is the magnitude of the charge located at $r_{i}$ with the width of the Gaussian $\xi_{i}$ given by

(17) $$\xi_{i}=\frac{\xi_{\text{Born}}}{R_{I}\sqrt{w_{i}}}$$

where, $w_{i}$ is the Lebedev weight referring to a unit sphere, and $R_{I}$ the radius of the sphere I on which the charge is placed. $\xi_{\text{Born}}$ is a dimensionless parameter, which depends on the Lebedev grid, and is adjusted to obtain the exact Born solvation energy for a conductor and a uniform surface charge distribution [63]. Within the Gaussian charge scheme, the off‐diagonal elements of the matrix A are the coulomb interactions of the Gaussians $g_{i}$ and $g_{j}$ and can be evaluated analytically via

(18) $$A_{\mathrm{ij}}=\frac{\mathrm{erf}\left(\xi_{\mathrm{ij}}r_{\mathrm{ij}}\right)}{r_{\mathrm{ij}}}$$

with

(19) $$\xi_{\mathrm{ij}}=\frac{\xi_{i}\xi_{j}}{\sqrt{\xi_{i}^{2}+\xi_{j}^{2}}}$$

and $r_{\mathrm{ij}}=∥r_{i}-r_{j}∥$. The diagonal elements of A contain the so‐called *self‐potential* and *self‐field* and are not easy to calculate, as they contain Coulomb interactions within discretized surface elements. As Gaussian charges remove the singularity in the Coulomb potential, the choice for the self‐potential interaction can be based on the limit
(20)

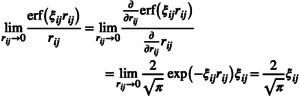

and a so‐called *switching function* $F_{i}$, to ensure continuity of the potential surface, consistent with References [60, 61, 64]. This defines $A_{\mathrm{ii}}$ as

(21) $$A_{\mathrm{ii}}=\frac{\xi_{i}\sqrt{\frac{2}{\pi }}}{F_{i}}$$

The switching function smoothly attenuates the contribution of the *i*th surface point to the solvation energy, as it passes into or out of the cavity. The improved switching function is calculated as a product of elementary switching functions $f\left(r_{i},R_{J},R_{J}\right)$ via

(22) $$F_{i}=\prod_{J,i\notin J}^{N}f\left(r_{i},R_{J},R_{J}\right)$$

where, N is the number of spheres that define the solute cavity and $R_{J}$ is the position vector of sphere J. The resulting approach using the improved switching function is called the improved switching Guassian approach (iSwiG) [62]. The elementary switching function is given by

(23) $$f\left(r_{i},R_{J},R_{J}\right)=1-\frac{1}{2}\left\{\mathrm{erf}\left[\xi_{i}\left(R_{J}-r_{\mathrm{iJ}}\right)\right]+\mathrm{erf}\left[\xi_{i}\left(R_{J}+r_{\mathrm{iJ}}\right)\right]\right\}$$

where, $r_{\mathrm{iJ}}=∥r_{i}-R_{J}∥$ is the distance between charge i and the center of sphere J.

### 2.4 Cavity Creation Scheme

The obtained draco radii are used to create a van der Waals (vdW) type cavity. This is done by using the external surface of the resulting overlapping spheres around each nucleus. Often, the used vdW radius is scaled by a factor, for example, 1.2, to mimic the impossibility of finite‐size solvent molecules entering possibly created crevices [60]. This is not necessary within the draco scheme as it is already included in the parameterization. In principle, a solvent probe can also be added to the radius of the vdW surface, creating either a solvent‐accessible surface (SAS) or a solvent‐excluded surface (SES). However, in this work, we only consider the vdW‐type cavity. The derivation for SAS‐ and SES‐type cavities is also possible and shown in, for example, References [61, 65].

For the numerical solution of the CPCM system of equations, a numerical integration grid is used. Typically, the number of grid points (charges) per sphere is fixed (isogrid). Generally, one could also employ a so‐called isodensity scheme. With this, a target density of charges per unit of surface area for each sphere is determined by finding the closest $n_{\mathrm{leb}}$ for each sphere via

(24) $$\rho_{n_{\mathrm{leb}}}=\frac{n_{\mathrm{leb},I}}{4\pi R_{I}^{2}}$$

**FIGURE 2:**
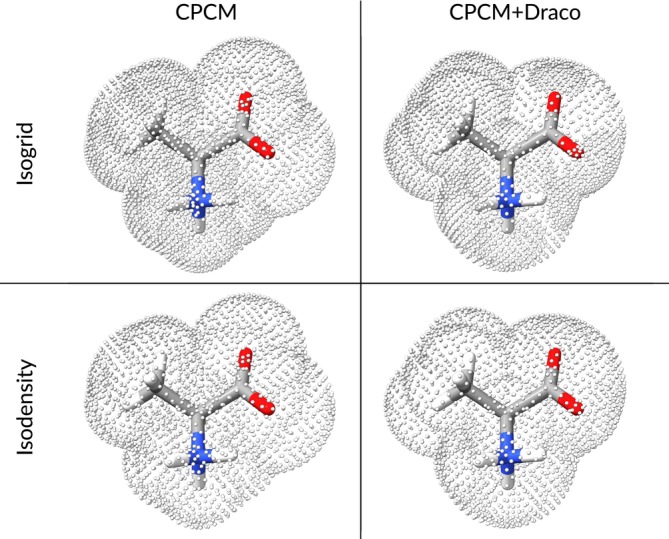
Comparison of the distribution of grid points (center of the Gaussian charges) on the cavity with the isogrid ($n_{\mathrm{leb}}=770$) and isodensity ($\rho_{n_{\mathrm{leb}}}=15\;{Å}^{-2}$) schemes without and with the draco scaling scheme for the zwitterionic form of alanine.

The two schemes are shown in Figure 2. An isodensity‐based scheme can adapt the integration grid more efficiently as radii change, which can be a potential advantage. However, for non‐static radii, the system‐dependent change of radii still risks introducing discontinuities in the potential energy surface, since the grid can change whenever a radius is modified. In either case, the grids are taken from Reference [63].

### 2.5 Derivation of the Gradient

The gradient is derived chronologically, starting with the energy expression of the polarization energy (15), and then proceeds step by step through the terms. The final expressions for all terms can be found in Appendix A. For the following sections, if the exact type of derivative is not specified, it refers to the derivative with respect to the nuclear displacement $R_{A}=\left\{x_{A},y_{A},z_{A}\right\}$.

In order to perform geometry optimizations with the iSwiG CPCM model, combined with nuclear coordinate‐dependent atomic radii, we need to differentiate the total energy w.r.t. to the perturbation $\frac{\partial }{\partial R_{A}}$. Inclusion of the CPCM model adds a polarization energy contribution to the total energy and thus requires the calculation of the electrostatic energy $E_{\mathrm{pol}}$ w.r.t. that perturbation. Applying the chain rule to the half‐product $\frac{1}{2}v^{⊤}\mathrm{Qv}$ yields the derivative of the polarization energy with respect to $R_{A}$, as shown in Equation (25).
(25)

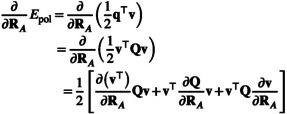




As Q is symmetric in CPCM, that is, $Q^{⊤}=Q$, the first and third terms are equivalent and allows us to rewrite Equation (25) as

(26) $$\frac{1}{2}\left[\frac{\partial \left(v^{⊤}\right)}{\partial R_{A}}\mathrm{Qv}+v^{⊤}\frac{\partial Q}{\partial R_{A}}v+v^{⊤}Q\frac{\partial v}{\partial R_{A}}\right]=\frac{1}{2}v^{⊤}\frac{\partial Q}{\partial R_{A}}v+v^{⊤}Q\frac{\partial v}{\partial R_{A}}$$

The symmetry of Q also allows the expression of Equation (9) as

(27) $$q=\mathrm{Qv}=v^{⊤}Q$$

and using the definition in the conductor‐like model of $Q=K^{-1}R$ (Equations 12 and 13) the gradient of the polarization energy can be expressed as
(28)

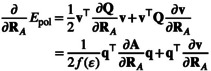




To calculate the gradient, the derivatives of A and v w.r.t. the nuclear displacements of atom A have to be found.

#### 2.5.1 Derivative of the Electrostatic Potential

The derivative of Equation (11) is given by

(29) $$\begin{array}{c} \frac{\partial v_{i}}{\partial R_{A}}=-\sum_{K=1}^{N_{\text{atoms}}}Z_{K}\left[\frac{1}{r_{\mathrm{iK}}^{3}}\left(r_{i}-R_{K}\right)\frac{\partial }{\partial R_{A}}\left(r_{i}-R_{K}\right)\right]\\ \;-\sum_{\mu \nu }P_{\mu \nu }\frac{\partial }{\partial R_{A}}\left(\left\langle \chi_{\mu }\left|\frac{1}{r_{i}-r}\right|\chi_{\nu }\right\rangle \right) \end{array}$$

and does not involve the calculation of $\frac{\partial P}{\partial R_{A}}$. This is because this derivative is already implicitly included in the term of the general SCF energy gradient that depends on the energy‐weighted density matrix (see Equation 51). Meanwhile, the nuclear contribution to $v_{i}$ depends on $\frac{\partial }{\partial R_{A}}\left(r_{i}-R_{K}\right)$, which we evaluate according to Equation (48). The last term of $\frac{\partial v_{i}}{\partial R_{A}}$ depends on $\frac{\partial }{\partial R_{A}}\left(\left\langle \chi_{\mu }\left|\frac{1}{r_{i}-r}\right|\chi_{\nu }\right\rangle \right)$, that is, the derivative of the electronic part of $v_{i}$ given by $\left\langle \chi_{\mu }\left|\frac{1}{r_{i}-r}\right|\chi_{\nu }\right\rangle$. This derivative is calculated via integration over primitive cartesian Gaussians $G_{a}$, which are one‐electron integrals of the type $\left\langle G_{a}\left|\frac{1}{r_{i}-r}\right|G_{b}\right\rangle$. These integrals can be expanded in Hermite expansion coefficients, $E_{t}^{\mathrm{ij}}$ and Hermite integrals $R_{\mathrm{tuv}}^{0}$ [66], which yields

(30) $$\left\langle G_{a}\left|\frac{1}{r_{i}-r}\right|G_{b}\right\rangle =\sum_{\mathrm{tuv}}E_{t}^{\mathrm{ij}}\left(R\right)E_{u}^{\mathrm{kl}}\left(R\right)E_{v}^{\mathrm{mn}}\left(R\right)R_{\mathrm{tuv}}^{0}\left(r_{i}\right)\equiv \sum_{\mathrm{tuv}}\text{EEER}$$

The derivative w.r.t. nuclear displacements of the electronic part of the potential, $v_{\mathrm{el}}$ (Equation 11) can be separated into two terms, $g_{1}$ and $g_{2}$, that depend on $\left(\frac{\partial \mathrm{EEE}}{\partial R_{A}}R\right)$ and $\left(\mathrm{EEE}\frac{\partial R}{\partial R_{A}}\right)$, respectively.

(31) $$\frac{\partial v_{\mathrm{el}}}{\partial R_{A}}=g_{1}\left(\frac{\partial \mathrm{EEE}}{\partial R_{A}}R\right)+g_{2}\left(\mathrm{EEE}\frac{\partial R}{\partial R_{A}}\right)$$

The term $g_{1}$ only depends on the position of the basis functions, which are atom centered, so it does not carry additional draco contributions. The second term, $g_{2}$, depends on the positions of the charges and can consequently be written as

(32) $$g_{2}\left(\mathrm{EEE}\frac{\partial R}{\partial R_{A}}\right)=\sum_{i}^{N_{q}}\left[\frac{\partial v_{\mathrm{el}}}{\partial x_{i}}\frac{\partial x_{i}}{\partial R_{A}}+\frac{\partial v_{\mathrm{el}}}{\partial y_{i}}\frac{\partial y_{i}}{\partial R_{A}}+\frac{\partial v_{\mathrm{el}}}{\partial z_{i}}\frac{\partial z_{i}}{\partial R_{A}}\right]$$

The partial derivatives $\frac{\partial r_{i}}{\partial R_{A}}$ are calculated following Section 2.5.6.

#### 2.5.2 Derivative of the A Matrix

The derivative of the diagonal elements of the matrix A (Equation 21) is given by
(33)

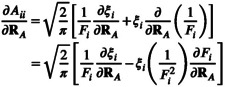

and requires the derivative of the Gaussian widths (Section 2.5.3) and switching function (Section 2.5.4). The gradient of the off‐diagonal elements of A (Equation 18) is given by
(34)

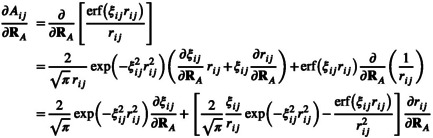

and requires the derivatives of the pair‐wise Gaussian width $\xi_{\mathrm{ij}}$ (Section 2.5.3) and distance $r_{\mathrm{ij}}$ (Section 2.5.6).

#### 2.5.3 Derivative of the Gaussian Widths

The derivatives of the Gaussian widths in Equations (17) and (19) are given by
(35)

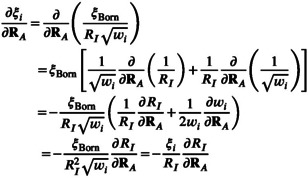

and
(36)

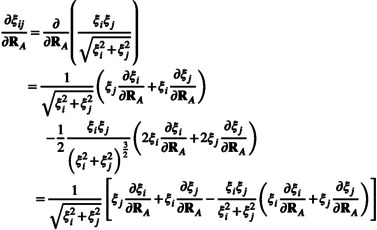

Since the Lebedev weights are effectively constants once a Lebedev quadrature grid is chosen for the unit sphere, the partial derivative $\frac{\partial w_{i}}{\partial R_{A}}$ is zero. The calculation of the derivatives of the Gaussian widths thus only requires the derivative of the radii given in Section 2.5.5.

#### 2.5.4 Derivative of the Switching Function

The derivative of $F_{i}$ is given by
(37)

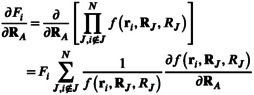

and the needed derivative of the elementary switching function f is given by
(38)

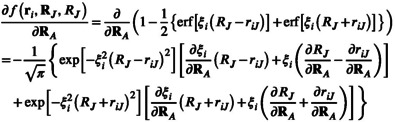

The derivative of the elementary switching function requires the partial derivatives of the Gaussian widths $\xi_{i}$ (Section 2.5.3), the radii $R_{J}$ (Section 2.5.5), and the distance between grid point and center of a sphere $r_{\mathrm{iJ}}$ (Section 2.5.6).

#### 2.5.5 Derivative of the Radii

The key change in the derivative of the CPCM energy is the non‐zero derivative of the radii w.r.t. to nuclear displacements, $\frac{\partial R_{I}}{\partial R_{A}}\neq 0$. Within the draco model this quantity is given by

(39) $$\begin{array}{c} \begin{array}{l} \frac{\partial R_{I}}{\partial R_{A}}=R_{I,0}\frac{\partial f_{I}}{\partial R_{A}}=R_{I,0}\frac{\partial }{\partial R_{A}}\left\{\mathrm{erf}\left[a_{Z_{I}}\left(q_{I,\mathrm{eff}}-b_{Z_{I}}\right)\right]+1\right\}\\ \;=R_{I,0}\frac{2}{\sqrt{\pi }}\exp\left\{-{\left[a_{Z_{I}}\left(q_{I,\mathrm{eff}}-b_{Z_{I}}\right)\right]}^{2}\right\}\left(a_{Z_{I}}\frac{\partial q_{I,\mathrm{eff}}}{\partial R_{A}}\right) \end{array} \end{array}$$

which yields

(40) $$\begin{array}{c} \begin{array}{l} \frac{\partial R_{I}}{\partial R_{A}}=\frac{2R_{I,0}a_{Z_{I}}}{\sqrt{\pi }}\exp\left\{-{\left[a_{Z_{I}}\left(q_{I,\mathrm{eff}}-b_{Z_{I}}\right)\right]}^{2}\right\}\\ \;\times \left[\frac{\partial q_{I}}{\partial R_{A}}\left(1+k_{Z_{I}}{\mathrm{CN}}_{I}\right)+q_{I}k_{Z_{I}}\frac{\partial {\mathrm{CN}}_{I}}{\partial R_{A}}\right] \end{array} \end{array}$$

To calculate Equation (40), the partial derivative of the atomic partial charges $\frac{\partial q_{I}}{\partial R_{A}}$ and the coordination number $\frac{\partial {\mathrm{CN}}_{I}}{\partial R_{A}}$ are required. The derivative of the charges depends on the model used. In this study the electronegativity‐equilibration (EEQ) charge model will be used. In this case, the gradient $\frac{\partial q_{I}}{\partial R_{A}}$ is that from Reference [57]. The partial derivative $\frac{\partial {\mathrm{CN}}_{I}}{\partial R_{A}}$ of the coordination number is given by

(41) $$\frac{\partial {\mathrm{CN}}_{I}}{\partial R_{A}}=-\sum_{I\neq J}^{N}\left(\frac{1}{f_{1}^{2}f_{2}}\frac{\partial f_{1}}{\partial R_{A}}+\frac{1}{f_{1}f_{2}^{2}}\frac{\partial f_{2}}{\partial R_{A}}\right)$$

and the derivative of the functions $f_{1}$ and $f_{2}$ by

(42) $$\frac{\partial f_{1}}{\partial R_{A}}=\frac{40}{3}\exp\left[-10\left(\frac{4}{3}\frac{R_{\mathrm{IJ},\mathrm{cov}}}{r_{\mathrm{IJ}}}-1\right)\right]\left[\frac{R_{\mathrm{IJ},\mathrm{cov}}}{r_{\mathrm{IJ}}^{3}}r_{\mathrm{IJ}}\cdot \left(\frac{\partial R_{I}}{\partial R_{A}}-\frac{\partial R_{J}}{\partial R_{A}}\right)\right]$$

and

(43) $$\begin{array}{c} \frac{\partial f_{2}}{\partial R_{A}}=20\exp\left[-20\left(\frac{\frac{4}{3}R_{\mathrm{IJ},\mathrm{cov}}+2}{r_{\mathrm{IJ}}}-1\right)\right]\\ \;\times \left[\frac{\frac{4}{3}R_{\mathrm{IJ},\mathrm{cov}}+2}{r_{\mathrm{IJ}}^{3}}r_{\mathrm{IJ}}\cdot \left(\frac{\partial R_{I}}{\partial R_{A}}-\frac{\partial R_{J}}{\partial R_{A}}\right)\right] \end{array}$$

with $r_{\mathrm{IJ}}=∥R_{I}-R_{J}∥$ and $R_{\mathrm{IJ},\mathrm{cov}}=R_{I,\mathrm{cov}}+R_{J,\mathrm{cov}}$ is the sum of the covalent radii of atom I and J as given in Reference [57].

#### 2.5.6 Derivative of the Nuclear and Grid Point Positions

Finally, we want to discuss the derivatives of $r_{i}$, $R_{I}$, $r_{\mathrm{ij}}$, and $r_{\mathrm{iJ}}$. For the regular CPCM gradient, the derivative of the positions of the grid points is given by

(44) $$\frac{\partial r_{i}}{\partial R_{A}}=\left\{\begin{array}{cc} 1 & \text{if}\;i\in A\\ 0 & \text{else} \end{array}\right.$$

and the derivative of the nuclear positions by

(45) $$\frac{\partial R_{I}}{\partial R_{A}}=\left\{\begin{array}{cc} 1 & \text{if}\;I=A\\ 0 & \text{else} \end{array}\right.$$

However, within the draco scheme, the radius of sphere I can change even if atom J is displaced. It follows that the position of i on sphere I can also change if J is moved and thus Equation (44) is not correct anymore. Introducing a unit vector $u_{i}$ which locates the *i*th point of a given Lebedev quadrature w.r.t. the center of its sphere I as

(46) $$u_{i}=\frac{r_{i}-R_{I}}{R_{I}}$$

yields a general definition of $r_{i}$ as

(47) $$r_{i}=R_{I}+R_{I}u_{i}$$

The final $\frac{\partial r_{i}}{\partial R_{A}}$ reads

(48) $$\frac{\partial r_{i}}{\partial R_{A}}=\left\{\begin{array}{cc} \frac{1}{R_{I}}\frac{\partial R_{I}}{\partial R_{A}}\left(r_{i}-R_{I}\right)+1 & \text{if}\;I=A\\ \frac{1}{R_{I}}\frac{\partial R_{I}}{\partial R_{A}}\left(r_{i}-R_{I}\right) & \text{else} \end{array}\right.$$

and it reduces to the result of Equation (44) if $\frac{\partial R_{I}}{\partial R_{A}}=0$. For the derivative of $r_{\mathrm{ij}}$ (and analogous $r_{\mathrm{iJ}}$ or $r_{\mathrm{IJ}}$), we apply the chain rule and obtain
(49)

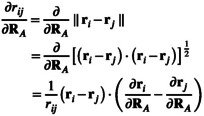




### 2.6 CPCM SCF Equations

The electrostatic solvation energy is included in the SCF total energy via

(50) $$\begin{array}{c} \begin{array}{l} \;E=\sum_{\mu \nu }\sum_{\sigma }P_{\mu \nu \sigma }h_{\mu \nu \sigma }\\ +\frac{1}{2}\sum_{\mu \nu }\sum_{\sigma \eta }\left(P_{\mu \nu \sigma }P_{\kappa \tau \eta }-a_{X}\delta_{\sigma \eta }P_{\mu \tau \sigma }P_{\nu \kappa \eta }\right)\left(\mu \nu |\kappa \tau \right)\\ \;+V_{\mathrm{NN}}+\frac{1}{2}q^{⊤}v+\sum_{\sigma }E_{\mathrm{XC}}\left[\rho_{\sigma }\left(r\right)\right] \end{array} \end{array}$$

where, $P_{\mu \nu \sigma }=\sum_{i}c_{\mathrm{\mu i\sigma}}^{*}c_{\mathrm{\nu i\sigma}}$ is the solute density matrix for spin $\sigma ,\eta =\left\{\alpha ,\beta \right\}$, with $c_{\mathrm{\mu i\sigma}}$ being the molecular orbital coefficients. The Mulliken notation is used for the two‐electron integrals over the atom‐centered basis functions $\left\{\mu \right\}$, and $a_{X}$ denotes the fraction of Hartree–Fock exchange. $V_{\mathrm{NN}}$ accounts for nuclear repulsion. The $\frac{1}{2}q^{⊤}v=E_{\mathrm{pol}}$ term describes the solvent‐solute interaction (Equation 15) and $E_{\mathrm{XC}}\left[\rho_{\sigma }\left(r\right)\right]$ is the exchange‐correlation energy with $\rho_{\sigma }\left(r\right)=\sum_{\mu \nu }P_{\mu \nu }\mu \left(r\right)\nu \left(r\right)$ being the electron density at position r with spin σ. The first derivative of E w.r.t. nuclear displacement $R_{A}$ is given by

(51) $$\begin{array}{c} \begin{array}{l} \;\frac{\partial E}{\partial R_{A}}=E^{R_{A}}=\sum_{\mu \nu \sigma }P_{\mu \nu \sigma }h_{\mu \nu \sigma }^{R_{A}}\\ \;+\frac{1}{2}\sum_{\text{μνση}}\left(P_{\mu \nu \sigma }P_{\kappa \tau \eta }-a_{X}\delta_{\sigma \eta }P_{\mu \tau \sigma }P_{\nu \kappa \eta }\right)\times {\left(\mu \nu |\kappa \tau \right)}^{R_{A}}\\ \;-\sum_{\mu \nu \sigma }S_{\mu \nu }^{R_{A}}W_{\mu \nu \sigma }+V_{\mathrm{NN}}^{R_{A}}+q^{⊤}v^{\left(R_{A}\right)}+\frac{1}{2f\left(\varepsilon \right)}q^{⊤}A^{R_{A}}q\\ \;+\sum_{\sigma }E_{\mathrm{XC}}^{R_{A}}\left[\rho_{\sigma }\left(R_{A}\right)\right] \end{array} \end{array}$$

where, S and W are the overlap and energy‐weighted density matrices, respectively. The enclosure of $R_{A}$ in parenthesis (i.e., 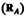) indicates that the derivative does not involve the calculation of the derivative of the density matrix P. Note that by definition W=PFP, with F being the Fock matrix. In the CPCM approach, a solvation contribution is additionally added to the Fock matrix elements over basis functions via

(52) $$\begin{array}{c} \begin{array}{l} F_{\mu \nu \sigma }=h_{\mu \nu \sigma }+\sum_{\kappa \tau \sigma }P_{\kappa \tau \sigma }\left[\left(\kappa \tau |\mu \nu \right)-a_{X}\delta_{\sigma \eta }\left(\kappa \nu |\mu \tau \right)\right]\\ \;+V_{\mu \nu \sigma }^{\mathrm{XC}}+q^{⊤}v_{\mu \nu \sigma } \end{array} \end{array}$$

where

(53) $$V_{\mu \nu \sigma }^{\mathrm{XC}}=\int \varphi_{\mu }\left(r\right)\frac{\partial E_{\mathrm{XC}}\left[\rho_{\sigma }\left(r\right)\right]}{\partial \rho_{\sigma }\left(r\right)}\varphi_{\nu }$$

is the exchange–correlation potential and $v_{\mu \nu \sigma }$ is the potential due to the shells $\chi_{\mu }$ and $\chi_{\nu }$ at the position at the charges.

### 2.7 Computational Details

All calculations were performed with a development version of ORCA 6.0.0 [54]. For the shown examples, the $r^{2}\text{SCAN}$‐based composite method $r^{2}\text{SCAN}‐3c$, and the range‐separated hybrid $\omega r^{2}\text{SCAN}$ [67, 68, 69, 70, 71, 72], with the respective def2‐mTZVPP and def2‐TZVPP basis sets are used [73, 74, 75, 76, 77]. For $\omega r^{2}\text{SCAN}$, the DFT‐D4 dispersion correction is used [57, 78, 79]. The gas‐phase geometries that give the starting point for our tests were obtained using the composite HF‐3c method [80]. The needed auxiliary basis sets are generated on the fly using *AutoAux* [81]. The *RIJCOSX* approximation is used for all hybrid calculations [82, 83, 84, 85]. The *ExtremeSCF* convergence criterion with the *DefGrid3* grid is used. The chosen SCF settings involve a tolerance of $1\times 10^{-14}$ a.u. for the change in energy, maximum density and RMS density, which means numerical precision. For geometry optimizations, we adopt the *VeryTightOpt* settings, which use an energy tolerance of $2\times 10^{-7}$ Eh, $3\times 10^{-5}$ a.u. for the maximum component of the gradient vector, $8\times 10^{-6}$ a.u. for the root mean square (rms) gradient, $2\times 10^{-4}$ a.u. for the maximum allowed displacement, and $1\times 10^{-4}$ a.u. for the maximum rms displacement. The numerical gradient is calculated using a four‐point central difference formula via

(54) $$\frac{\partial E}{\partial R}=\frac{1}{12\delta r}\left[-E\left(r+2\delta r\right)+8E\left(r+\delta r\right)-8E\left(r-\delta r\right)+E\left(r-2\delta r\right)\right]$$

and a step size of $\delta =5.0\times 10^{-3}$ bohr without the use of translational invariance (*TransInvar* set to false). The isogrid scheme is used for all calculations with $n_{\mathrm{leb}}=194$. The isodensity scheme uses the value of $\rho_{n_{\mathrm{leb}}}=7.5\;{Å}^{-2}$. The unscaled radii in Equation (1) are used as described in Reference [86]. The improved switching/Gaussian approach is used for both models [60, 61, 62, 86].

## 3 Results and Discussion

All tests will be conducted using water as the solvent, as it offers a large ε and therefore a strong influence of the solvation models on the electronic structure. The draco scheme will also use charges from the EEQ model, if not specifically noted otherwise.

### 3.1 Geometry Optimizations

**FIGURE 3:**
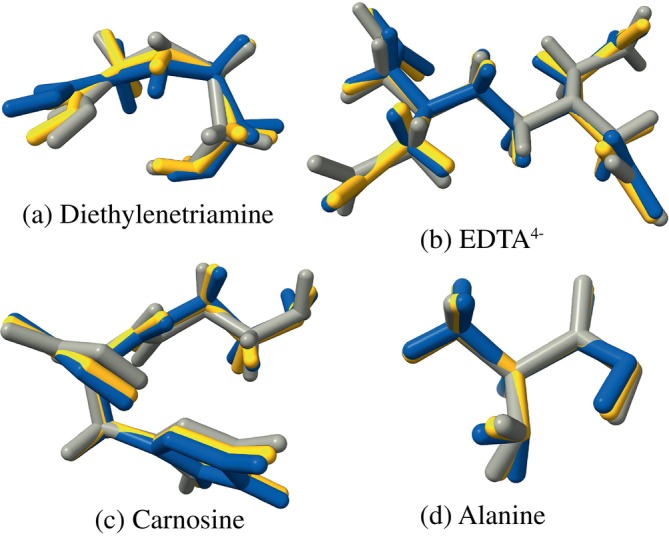
Superimposed optimized geometries of the initial HF‐3c gas‐phase geometry (gray, 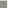) the CPCM (yellow, 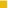), and CPCM+draco (blue, 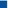) models with water as a solvent.

**FIGURE B1:**
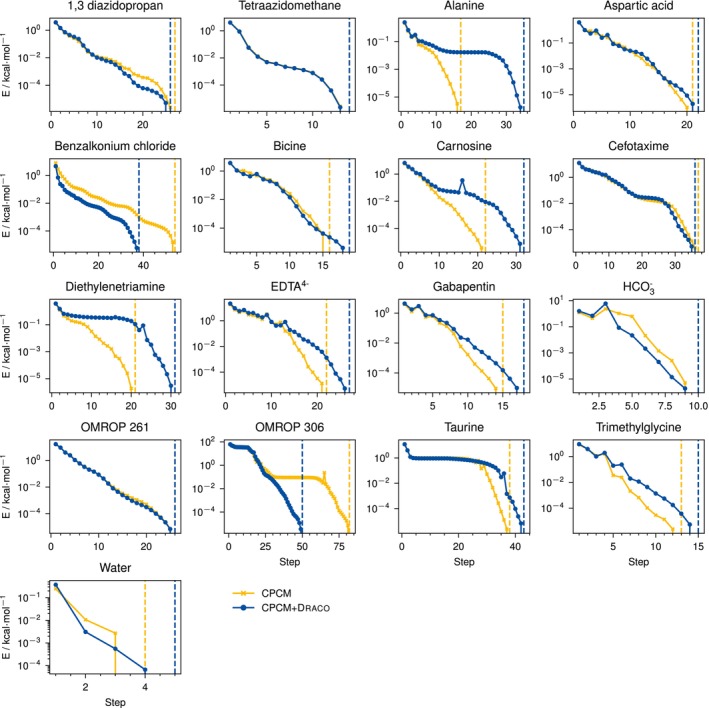
Optimization energy profiles (i.e., energy during the optimization as a function of the steps) of CPCM and CPCM+draco for all structures using a logarithmic scale in kcal mol^−1^. The final energy is set so zero. The dashed line indicates the point of convergence for both respective models.

As a first test, various geometries of organic and metal–organic molecules are optimized (using the analytical gradient), starting from an HF‐3c optimized gas‐phase geometry to provide a suboptimal (in the context of solvation) starting geometry. The test set includes smaller and larger organic molecules (e.g., Cefotaxime), ionic and zwitterionic compounds (e.g., benzalkonium chloride), and transition metal complexes (like the structures from the OMROP [87, 88] benchmark set). Some of the examples are shown in Figure 3. The geometry rmsd (calculated using quaternions as in Reference [89]) between the optimized structures of the two models w.r.t. each other and for each model w.r.t. the initial HF‐3c gas‐phase geometry is given. Additionally, the numerical and analytical gradients of the starting HF‐3c geometry are compared, that is, the gradient of the first step of the geometry optimization. The results are given in Table 1 with the most differing geometries given in Figure 3. The energy profiles of the optimizations are given in Appendix B, Figure B1.

**TABLE 1:** Root mean square (rms) and maximum (max) of the absolute difference between the analytical and numerical gradient (|ΔG|=|Δ(∇E)| with $\Delta (\;\nabla E)={\left(\nabla E\right)}^{\text{numeric}}-{\left(\nabla E\right)}^{\text{analytic}}$) of the initial HF‐3c gas‐phase geometry in atomic units for the total energy gradient.

Structure	CPCM			CPCM+draco			rmsd/Å		
	rms/a.u.	max/a.u.	*n*_opt_	rms/a.u.	max/a.u.	*n*_opt_	CPCM/CPCM+draco	CPCM/HF‐3c	CPCM+draco/HF‐3c
1,3‐Diazidopropan	1.1 × 10^−8^	2.4 × 10^−8^	27	1.1 × 10^−8^	2.5 × 10^−8^	26	0.022	0.243	0.234
Alanine	1.3 × 10^−8^	2.5 × 10^−8^	17	1.3 × 10^−8^	2.7 × 10^−8^	35	0.200	0.121	0.310
Aspartic acid	1.3 × 10^−8^	2.5 × 10^−8^	21	1.3 × 10^−8^	2.6 × 10^−8^	22	0.017	0.137	0.144
Benzalkonium chloride	1.1 × 10^−8^	2.5 × 10^−8^	54	1.2 × 10^−8^	2.9 × 10^−8^	38	0.174	0.217	0.243
Bicine	1.1 × 10^−8^	2.1 × 10^−8^	16	1.1 × 10^−8^	2.1 × 10^−8^	19	0.033	0.130	0.110
Carnosine	1.1 × 10^−8^	2.3 × 10^−8^	22	1.1 × 10^−8^	2.3 × 10^−8^	32	0.233	0.429	0.646
Cefotaxime	1.2 × 10^−8^	3.3 × 10^−8^	37	1.7 × 10^−8^	8.4 × 10^−8^	36	0.148	1.721	1.820
Diethylenetriamine	1.0 × 10^−8^	2.1 × 10^−8^	21	1.2 × 10^−8^	3.3 × 10^−8^	31	0.417	0.389	0.700
EDTA^4−^	1.1 × 10^−8^	2.7 × 10^−8^	22	1.2 × 10^−8^	2.8 × 10^−8^	27	0.184	0.436	0.501
Gabapentin	1.1 × 10^−8^	2.5 × 10^−8^	15	1.2 × 10^−8^	2.8 × 10^−8^	18	0.030	0.086	0.100
HCO_3_^−^	8.0 × 10^−9^	1.4 × 10^−8^	10	7.8 × 10^−9^	1.4 × 10^−8^	10	0.004	0.026	0.030
OMROP‐261	1.3 × 10^−8^	3.2 × 10^−8^	26	1.3 × 10^−8^	2.8 × 10^−8^	26	0.013	0.150	0.141
OMROP‐306	1.3 × 10^−8^	4.2 × 10^−8^	82	1.0 × 10^−8^	2.0 × 10^−8^	50	0.168	0.736	0.713
Taurine	9.9 × 10^−9^	2.3 × 10^−8^	38	9.7 × 10^−9^	2.2 × 10^−8^	43	0.059	0.301	0.264
Tetraazidomethane	1.1 × 10^−8^	2.9 × 10^−8^	14	1.1 × 10^−8^	2.4 × 10^−8^	14	0.006	0.127	0.126
Trimethylglycine	9.4 × 10^−9^	2.0 × 10^−8^	13	9.5 × 10^−9^	2.0 × 10^−8^	15	0.037	0.116	0.122
Water	1.5 × 10^−8^	2.5 × 10^−8^	4	1.7 × 10^−8^	3.3 × 10^−8^	5	0.004	0.016	0.016
Mean	1.1 × 10^−8^	2.5 × 10^−8^	26	1.2 × 10^−8^	2.8 × 10^−8^	26	0.103	0.317	0.366

*Note:* The total number of needed steps in the optimization is given as $n_{\mathrm{opt}}$. The rmsd shows the deviation of the CPCM and CPCM+draco final geometries w.r.t. to each other, and the final CPCM and CPCM+draco geometry w.r.t. the initial HF‐3c geometry in Å.

Comparisons of numerical and analytical gradients for CPCM+draco reveal the same excellent level of agreement as for regular CPCM. Across the test set, the maximum deviation between numerical and analytical gradients is on the order of $10^{-8}$ a.u., and both the mean of the maximum absolute deviation and the rms deviation remain virtually unchanged compared to standard CPCM. Although computing the numerical gradient requires reconstructing the cavity four times per displacement—thereby slightly altering the number of charges each time—this introduces no additional errors, even in the draco case where the radii also vary additionally.

The final geometries of the CPCM and CPCM+draco models are found to be very similar with the rmsd between the models being well below 1 Å. The mean rmsd between the model's final geometry and the initial geometry is, however, larger for CPCM+draco (0.37 Å), meaning that the CPCM+draco PES differs more compared to HF‐3c gas‐phase than CPCM (0.32 Å). The largest differences between the CPCM and CPCM+draco models can be found for diethylenetriamine, taurine, carnosine, and EDTA^4−^. The resulting geometries of these are shown in Figure 3, including the respective initial HF‐3c geometries, where it is obvious that the final CPCM+draco structures differs more from the initial geometry (as also visible in the respective rmsds). For these mentioned structures, draco is expected to deliver different results as it changes the radii more drastically for heteroatoms and strongly partially charged atoms [51].

On average, both models need around 26 steps to converge the optimization across the test set. Molecules like benzalkonium chloride and OMROP 306 require more steps with plain CPCM, while alanine and carnosine (Figure 3c,d) require more with CPCM+draco. In all of these cases, the extra steps correlate with a more pronounced structural change compared with the initial geometry—that is, the final geometry for that method ends up to be more different from the initial structure and from the other method's result.

The energy‐optimization profiles (Appendix B, Figure B1) reflect the same trend. Most systems converge at similar rates, but those with final geometries that deviate more strongly require additional steps. The notable exceptions (where one approach requires more steps) correlate again with those systems for which the two methods yield more differing final geometries. Notably, though CPCM+draco often leads to a geometry further away from the HF‐3c starting structure (compared to CPCM), it does so without significantly increasing the overall convergence time (i.e., number of steps). Thus, despite the altered radii and thus modified PES, CPCM+draco remains just as efficient on average as plain CPCM on our test set.

### 3.2 Continuity of the Potential Energy Surface

**FIGURE 4:**
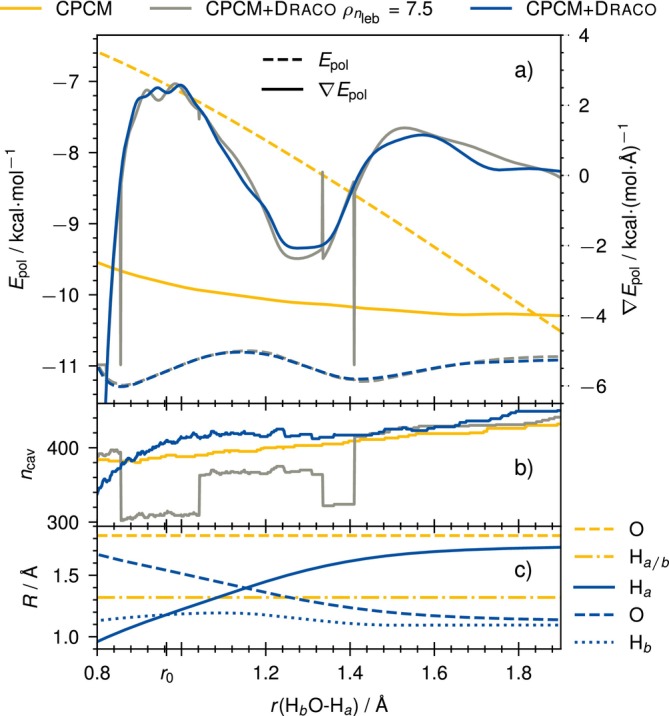
Abstraction of H+ from water, calculated at $r^{2}\text{SCAN}‐3c$ level. The CPCM (yellow) and CPCM+draco (blue) models both use the isogrid scheme with $n_{\mathrm{leb}}=194$, whereas the CPCM+draco $\rho_{n_{\mathrm{leb}}}=7.5$ uses the isodensity scheme (7.5 charges per ${Å}^{2}$). Subplot (a) shows the model's solvation energy and its gradient, (b) shows the respective total number of grid points, and (c) shows the respective used radii for the CPCM+draco approaches (blue) and static CPCM radii (yellow). The blue line represents both the isogrid and isodensity approaches for CPCM+draco, as they are identical. The occurrence of a change in Lebedev grid for CPCM+draco using the isodensity scheme is visible by the discontinuities in (b).

To show the continuity of the potential energy surface of the CPCM+draco model and its gradient, we provide the testcase of the abstraction of H+ from water, effectively forming

$$\mathrm{HO}‐H\to {\mathrm{HO}}^{\delta^{-}}\cdots H^{\delta^{+}}$$

from 0.8× to 2.0× of the equilibrium bond distance in $\mathrm{\delta r}\approx 9\times 10^{-4}\;Å$ steps. We investigate the polarization energy ($E_{\mathrm{pol}}$, Figure 4a) and its gradient ($\nabla E_{\mathrm{pol}}$, Figure 4a), while simultaneously showing the total number of grid points on the cavity ($n_{\mathrm{cav}}$, Figure 4b) and the radii of all species in the molecule (R, Figure 4c). For the methods, we investigate CPCM and CPCM+draco (yellow and blue) with the isogrid scheme and additionally include CPCM+draco with the isodensity scheme (gray). The result is given in Figure 4.

Comparing CPCM with CPCM+draco using the isogrid scheme we find differing PES and thus also gradients. This arises from the draco‐based adjustments to the atomic radii (Figure 4c), which vary substantially during the abstraction and thus significantly change the respective potential energy surface. The radius of the abstracted hydrogen increases while the radius of the oxygen decreases similarly. It should be kept in mind that the draco model is originally parametrized only using equilibrium geometries of small and simple organic molecules [51, 90]. In any case, however, we find a smooth and continuous PES with CPCM+draco making geometry optimizations easily possible.

For using CPCM+draco, we find that the choice of the cavity discretization scheme (isogrid or isodensity scheme) has a critical impact on the continuity of the potential energy surface. For the isodensity scheme, we observe problematic discontinuities in the PES: as the radii change, the number of grid points also changes abruptly (Figure 4b), causing a slight but sudden energy jump and thus a discontinuous potential energy surface. This can be seen at, for example, 0.9, 1.0, 1.3, and 1.4Å and is especially visible in the gradient. This renders the usage of the isodensity scheme for CPCM+draco unsuitable for a whole geometry optimization, which is why we recommend to employ per default a scheme where the number of charges per sphere in the cavity remains constant. This would be the case for (i), the isogrid scheme or (ii), the isodensity scheme only applied to the initial geometry—keeping the resulting number of charges for each sphere constant for the next geometry optimization steps.

### 3.3 Numerical Hessian: Vibrational Spectra and Transitions States

**FIGURE 5:**
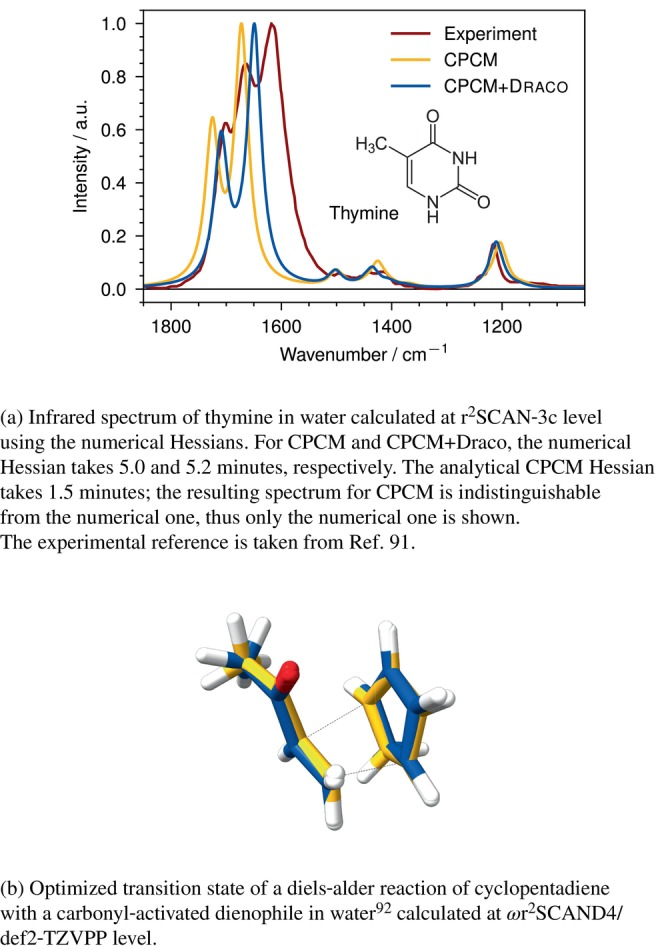
Examples of an IR spectrum and transition‐state optimization using the CPCM (yellow, 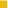) and CPCM+draco (blue, 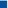) models. CPCM uses the analytical Hessian, whereas CPCM+draco uses the numerical Hessian.

We would like to highlight that even though the Hessian is not implemented analytically for CPCM+draco, the analytic gradient already allows the calculation of the numerical Hessian in a reasonable time. This enables, for example, the calculation of transition states, as shown for a Diels‐Alder reaction in water in Figure 5b [91], or IR spectra as shown for thymine in water in Figure 5a. For these exemplary calculations, we employ the quasi‐rigid‐rotor‐harmonic‐oscillator (RRHO) approximation [92].

Comparing the calculated IR spectra with the experiment [93], we observe that CPCM+draco generally aligns better with the experiment, particularly in the 1600–1700 cm^−1^ range and at the peak at 1200 cm^−1^. The relative intensities predicted by CPCM+draco also show better agreement, especially in the 1400–1500 cm^−1^ range. It is worth mentioning, that for the numerical Hessian, the cavity has to be produced for each displacement. This potentially adds noise w.r.t. the analytical Hessian and thus can lead to slightly different results.

### 3.4 Timings

**FIGURE 6:**
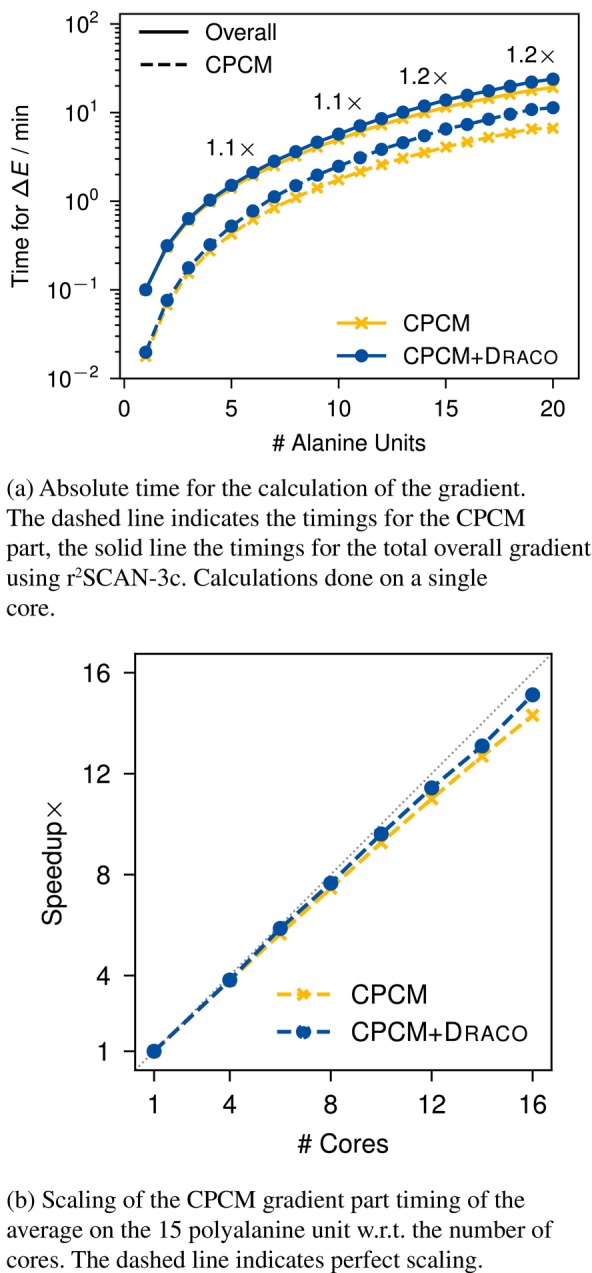
Timings for the gradient calculation of a polyalanine chain. The numbers indicate the ratio between the CPCM+draco and CPCM gradient. The timings are an average obtained from n=10 calculations, done on an AMD EPYC 7763 processor.

The timings for the gradient are evaluated using a linear chain of alanine units taken from Reference [94]. The results are shown in Figure 6, which the scaling w.r.t. to system size is shown in Figure 6a and the scaling w.r.t. to the number of cores used for the calculation in Figure 6b.

Although the CPCM part of the gradient is noticeably more expensive by using draco (dashed line in Figure 6a)—especially for large systems—in the overall timings (i.e., the gradient of the total energy, solid line in Figure 6a), this is only slightly noticeable. For molecules smaller than 100 atoms (i.e., ≈10 alanine units), this is negligible as shown for $r^{2}\text{SCAN}‐3c$, with an increase in computational time per gradient evaluation of around 10%. In the context of a geometry optimization, the impact is even smaller, since the SCF has to be solved beforehand for every geometry step. The newly implemented CPCM+draco gradient actually scales slightly better with respect to the number of cores (Figure 6b) because the extra gradient terms introduced by draco can be parallelized more effectively, improving overall parallel efficiency.

## 4 Conclusions

We derived and implemented the analytical first derivatives of the SCF energy for the Gaussian‐switching CPCM model combined with nonstatic, atomic coordinate‐dependent radii. This is explicitly shown using the draco scheme. The draco scheme generates system‐ and atom‐specific radii, which are used to create the vdW‐cavity for the CPCM model. The use of the draco scheme has already been shown to greatly enhance the results combined with CPCM and SMD. In this work, we go through the theory and derivation in detail, which simplifies the implementation and also the combination with other radii scaling schemes in the future.

Comparison of the derived, implemented gradient with its numerical counterpart confirms excellent agreement, mirroring the result of regular CPCM. We further validated the derivation and implementation on a broad range of molecular systems, from small organic molecules to larger molecules and metal–organic complexes. In most cases, CPCM+draco yields geometries very similar to those obtained with conventional CPCM; only strongly polar, large structures show notable deviations, reflecting the different PES of CPCM+draco. Finally, we confirmed the continuity of the potential energy surface by examining a H+ abstraction from water—an example in which the draco radii change substantially—yet the resulting PES remained consistent and well‐behaved. Furthermore, we evaluated the computational efficiency of the CPCM+draco gradient. Timings for gradient calculations, especially on larger systems, showed a slight increase in computation time of the non‐static radii gradient. Due to the significantly larger number of terms in the resulting gradient, the timings are correspondingly longer. However, in geometry optimizations, this increase in time for the CPCM gradient part is hardly noticeable overall, as the remaining terms (e.g., XC gradient) are much more expensive terms. As our implementation scales even better with the number of cores used compared to the regular CPCM model, it is equally as suitable for the optimization of very large systems.

Implemented within the ORCA program suite, this work is freely available for academic use and allows for efficient geometry optimizations using the improved CPCM+draco and SMD+draco approaches.

## Conflicts of Interest

M.G.‐R. and C.R. are affiliated with FAccTs GmbH which licenses the ORCA programme package for commercial use.

## Data Availability

Data sharing not applicable to this article as no datasets were generated or analysed during the current study.

## Appendix A Final Gradient Terms

(A1)
$$\begin{array}{c} \begin{array}{c} \;\frac{\partial A_{\mathrm{ii}}}{\partial R_{A}}=\sqrt{\frac{2}{\pi }}\frac{1}{F_{i}}\frac{\partial \xi_{i}}{\partial R_{A}}\\ \;+\frac{\sqrt{2}}{\pi }\frac{\xi_{i}}{F_{i}}\sum_{J,i\notin J}^{N}\frac{1}{f\left(r_{i},R_{J},R_{J}\right)}\left\{\left(R_{J}+r_{\mathrm{iJ}}\right)\exp\left[-\xi_{i}^{2}{\left(R_{J}+r_{\mathrm{iJ}}\right)}^{2}\right]\right.\\ \;\left.+\left(R_{J}-r_{\mathrm{iJ}}\right)\exp\left[-\xi_{i}^{2}{\left(R_{J}-r_{\mathrm{iJ}}\right)}^{2}\right]\right\}\frac{\partial \xi_{i}}{\partial R_{A}}\\ \;+\frac{\sqrt{2}}{\pi }\frac{\xi_{i}^{2}}{F_{i}}\sum_{J,i\notin J}^{N}\frac{1}{f\left(r_{i},R_{J},R_{J}\right)}\left\{\exp\left[-\xi_{i}^{2}{\left(R_{J}+r_{\mathrm{iJ}}\right)}^{2}\right]\right.\\ \;\left.+\exp\left[-\xi_{i}^{2}{\left(R_{J}-r_{\mathrm{iJ}}\right)}^{2}\right]\right\}\frac{\partial R_{J}}{\partial R_{A}}\\ \;+\frac{\sqrt{2}}{\pi }\frac{\xi_{i}^{2}}{F_{i}}\sum_{J,i\notin J}^{N}\frac{1}{f\left(r_{i},R_{J},R_{J}\right)r_{\mathrm{iJ}}}\left\{\exp\left[-\xi_{i}^{2}{\left(R_{J}+r_{\mathrm{iJ}}\right)}^{2}\right]\right.\\ \;\left.-\exp\left[-\xi_{i}^{2}{\left(R_{J}-r_{\mathrm{iJ}}\right)}^{2}\right]\right\}\left(r_{i}-R_{J}\right)\cdot \left(\frac{\partial r_{i}}{\partial R_{A}}-\frac{\partial R_{J}}{\partial R_{A}}\right) \end{array} \end{array}$$

(A2)
$$\begin{array}{c} \begin{array}{l} \;\frac{\partial \xi_{i}}{\partial R_{A}}=-\frac{2R_{I,0}a_{Z_{I}}\xi_{i}}{\sqrt{\pi }}\left\{\frac{1}{R_{I}}\exp\left[-{\left(a_{Z_{I}}\left(q_{I,\mathrm{eff}}-b_{Z_{I}}\right)\right)}^{2}\right]\right.\\ \;\left.\times \left[\frac{\partial q_{I}}{\partial R_{A}}\left(1+k_{Z_{I}}{\mathrm{CN}}_{I}\right)+k_{Z_{I}}q_{I}\frac{\partial {\mathrm{CN}}_{I}}{\partial R_{A}}\right]\right\}-\frac{\xi_{i}}{2w_{i}}\frac{\partial w_{i}}{\partial R_{A}} \end{array} \end{array}$$

(A3)
$$\begin{array}{c} \begin{array}{l} \frac{\partial A_{\mathrm{ij}}}{\partial R_{A}}=\frac{2}{\sqrt{\pi }}\exp\left(-\xi_{\mathrm{ij}}^{2}r_{\mathrm{ij}}^{2}\right)\frac{\partial \xi_{\mathrm{ij}}}{\partial R_{A}}\\ +\left[\frac{2}{\sqrt{\pi }}\frac{\xi_{\mathrm{ij}}}{r_{\mathrm{ij}}^{2}}\exp\left(-\xi_{\mathrm{ij}}^{2}r_{\mathrm{ij}}^{2}\right)-\frac{\mathrm{erf}\left(\xi_{\mathrm{ij}}r_{\mathrm{ij}}\right)}{r_{\mathrm{ij}}^{3}}\right]\left(r_{i}-r_{j}\right)\cdot \left(\frac{\partial r_{i}}{\partial R_{A}}-\frac{\partial r_{j}}{\partial R_{A}}\right) \end{array} \end{array}$$

(A4)
$$\begin{array}{c} \begin{array}{l} \;\frac{\partial \xi_{\mathrm{ij}}}{\partial R_{A}}=\frac{1}{\sqrt{\xi_{i}^{2}+\xi_{j}^{2}}}\\ \;\times \left[\xi_{j}\frac{\partial \xi_{i}}{\partial R_{A}}+\xi_{i}\frac{\partial \xi_{j}}{\partial R_{A}}-\frac{\xi_{i}\xi_{j}}{\xi_{i}^{2}+\xi_{j}^{2}}\left(\xi_{i}\frac{\partial \xi_{i}}{\partial R_{A}}+\xi_{j}\frac{\partial \xi_{j}}{\partial R_{A}}\right)\right] \end{array} \end{array}$$
